# Tin Bromido Aluminate Networks with Bright Luminescence

**DOI:** 10.1002/open.202200226

**Published:** 2023-02-22

**Authors:** Silke Wolf, Ralf Köppe, Peter W. Roesky, Claus Feldmann

**Affiliations:** ^1^ Institut für Anorganische Chemie Karlsruhe Institute of Technology (KIT) Engesserstrasse 15 76131 Karlsruhe Germany

**Keywords:** emission quantum yield, ionic liquid, luminescence, network structure, tin bromido aluminates

## Abstract

The novel tin bromido aluminates [Sn_3_(AlBr_4_)_6_](Al_2_Br_6_) (**1**), Sn(AlBr_4_)_2_ (**2**), [EMIm][Sn(AlBr_4_)_3_] (**3**) and [BMPyr][Sn(AlBr_4_)_3_] (**4**) ([EMIm]: 1‐ethyl‐3‐methylimidazolium, [BMPyr]: 1‐butyl‐1‐methyl‐pyrrolidinium), are obtained from a ionic‐liquid‐based reaction of AlBr_3_ and SnCl_2_ or SnBr_2_, resulting in colorless and transparent crystals. **1** contains a neutral, inorganic _∞_^3^[Sn_3_(AlBr_4_)_6_] network filled with intercalated Al_2_Br_6_ molecules. **2** represents a 3D structure isotypic to Pb(AlCl_4_)_2_ or α‐Sr[GaCl_4_]_2_. **3** and **4** exhibit infinite _∞_^1^[Sn(AlBr_4_)_3_]^n−^ chains that are separated by the voluminous [EMIm]^+^/[BMPyr]^+^ cations. All title compounds contain Sn^2+^ coordinated by AlBr_4_ tetrahedra, resulting in chains or 3D networks. Moreover, all title compounds show photoluminescence due to Br^−^→Al^3+^ ligand‐to‐metal charge‐transfer excitation, followed by 5*s* ^2^ *p* ^0^←5*s* ^1^ *p* ^1^ emission on Sn^2+^. Most surprisingly, the luminescence is highly efficient (quantum yield >50 %). Specifically, **3** and **4** exhibit outstanding quantum yields of 98 and 99 %, which are the highest values observed for Sn^2+^‐based luminescence so far. The title compounds have been characterized by single‐crystal structure analysis, elemental analysis, energy‐dispersive X‐ray analysis, thermogravimetry, infrared and Raman spectroscopy, UV‐Vis and photoluminescence spectroscopy.

## Graphical abstract

**The tin bromido aluminates** [Sn_3_(AlBr_4_)_6_](Al_2_Br_6_), Sn(AlBr_4_)_2_, [EMIm][Sn(AlBr_4_)_3_] and [BMPyr][Sn(AlBr_4_)_3_] are prepared in ionic liquids. Some exhibit 3D network structures and bright Sn^2+^‐based photoluminescence with quantum yields near 100 %.

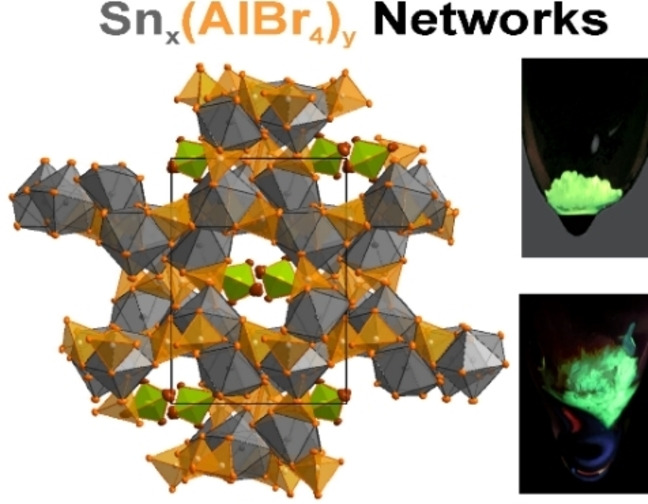

## Introduction

Sn^2+^‐based luminescent materials are typically less efficient in terms of quantum yield (QY) and light output (LO) as compared to, for instance, Mn^2+^‐based luminescent materials or lanthanide‐doped materials (e. g., with Eu^2+^, Eu^3+^, Tb^3+^).^[1]^ This lower performance can be related to the 5*s* ^2^ *p* ^0^↔5*s* ^1^ *p* ^1^ transition on Sn^2+^ and the strong coupling of both *s* and *p* orbitals with the lattice. In the case of tin, lattice coupling is much stronger than for the respective *d*↔*d*, *d*↔*f*, and *f*↔*f* transitions on transition metal cations or lanthanide metal cations.^[1]^ Due to lattice strong coupling, *s*↔*p* transitions are more sensitive to loss processes (e. g., lattice vibrations, lattice defects, impurities), show larger Stokes shifts and broader peak widths, and thus, a generally lower efficiency.^[1]^ Consequently, the number of efficient Sn^2+^‐based luminescent materials is low with, for instance, Ba_2_Li_2_Si_2_O_7_ : Sn^2+^, *M*_3_(PO_4_)_2_ : Sn^2+^ (M: Mg, Ca, Sr, Ba), or Ca_5_(PO_4_)_3_F : Sn^2+^ having quantum yields up to 85 % at maximum.[^2^, ^3^] These phosphors contain Sn^2+^ with concentrations of 1–10 mol− %, since concentration quenching is usually observed for higher Sn^2+^ concentrations (>10 mol− %).[^1^, ^2^] In addition to these conventional phosphors, organic‐inorganic hybrid tin halides have entered the literature since 2019 in the wake of methylammonium lead iodide perovskite as a novel solar absorber.^[4]^ Here, efficient fluorescence has been reported, particularly for hybrid tin bromides and iodides. Due to the small bandgaps of these compounds (e. g., [C_8_H_17_NH_3_]_2_[SnBr_4_], [(PEA)_4_SnBr_6_][(PEA)Br] ⋅ (CCl_2_H_2_)_2_ with PEA: phenylethylammonium), however, luminescence usually originates from semiconductor‐type valence band to conduction band transitions, superimposing the *s*–*p* transition of Sn^2+^.^[5]^

Using ionic‐liquid‐based synthesis, we could recently prepare the chlorido metallates [BMIm][Sn_3_Cl_7_] as well as [BMIm][Sn(AlCl_4_)_3_] and [BMPyr][Sn(AlCl_4_)_3_] ([BMIm]: 1‐butyl‐3‐methylimidazolium, [BMPyr]: 1‐butyl‐1‐methyl‐pyrrolidinium), which contain _∞_^2^[Sn_3_Cl_7_]^−^ zigzag‐planes as well as _∞_^1^[Sn(AlCl_4_)_3_]^−^ chains.^[6]^ Although they contain Sn^2+^ as a regular lattice constituent with molar quantities (instead of a dopant in a non‐luminescent lattice), they exhibit efficient luminescence with high quantum yields of 46, 51 and 76 %, respectively. This finding and specifically the missing concentration quenching can be ascribed to the large distances between the luminescent Sn^2+^ centers (≥386 pm in [BMIm][Sn_3_Cl_7_], ≥647 pm in [BMPyr][Sn(AlCl_4_)_3_]).^[6]^ Regarding these promising luminescence features, even more efficient emission and higher quantum yields could be expected for heavier halides with lower vibrational losses and more rigid network structures. With [Sn_3_(AlBr_4_)_6_](Al_2_Br_6_) (**1**), Sn(AlBr_4_)_2_ (**2**), [EMIm][Sn(AlBr_4_)_3_] (**3**) and [BMPyr][Sn(AlBr_4_)_3_] (**4**) ([EMIm]: 1‐ethyl‐3‐methylimidazolium), we now obtained four novel tin bromido aluminates with network structures showing extraordinary quantum yields up to 99 %.

## Results and Discussion

### Ionic‐liquid‐based synthesis

**Figure 1:**
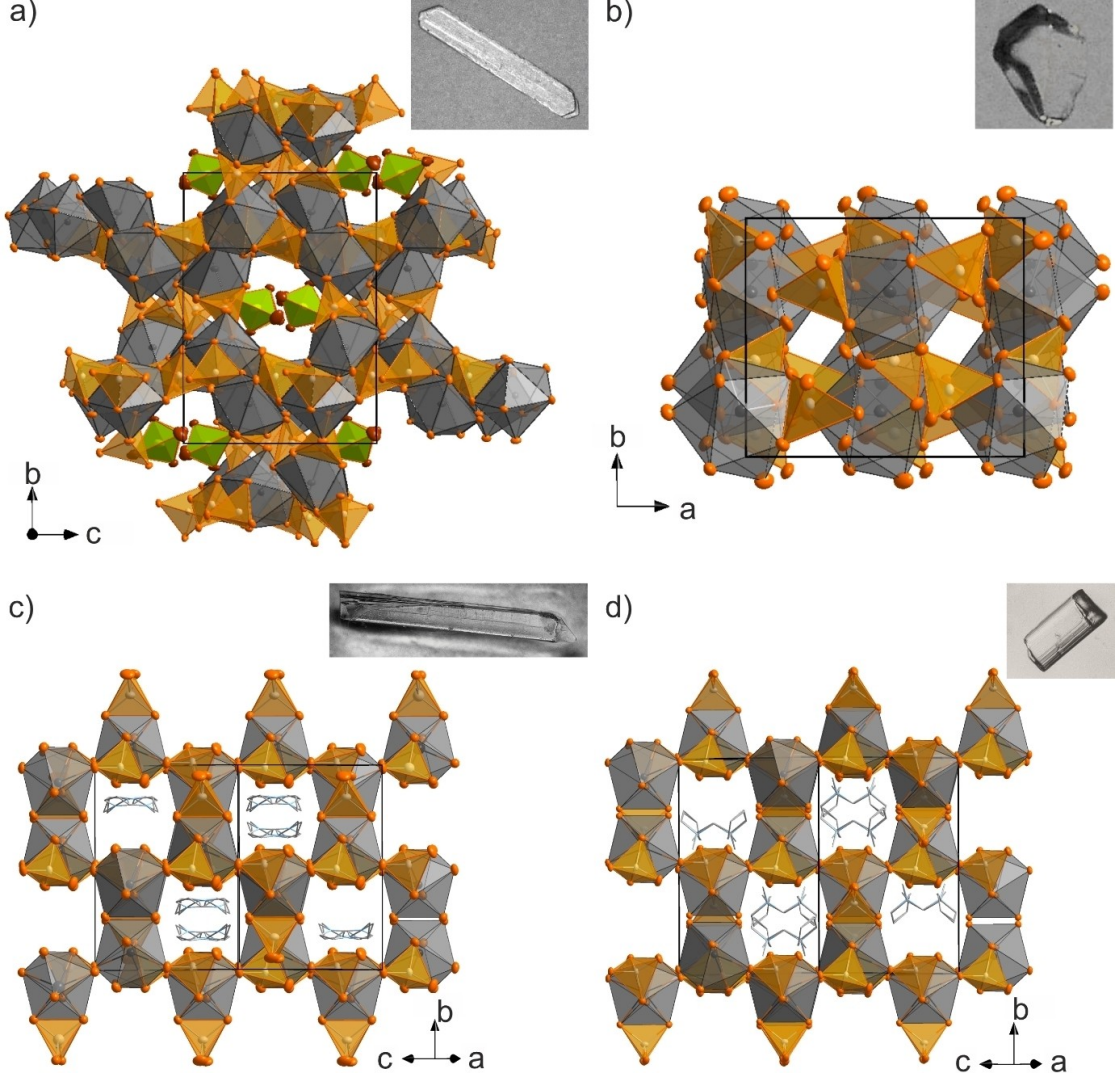
Unit cells and photos of single crystals of a) [Sn_3_(AlBr_4_)_6_][Al_2_Br_6_] (**1**), b) Sn(AlBr_4_)_2_ (**2**), c) [EMIm][Sn(AlBr_4_)_3_] (**3**), d) [BMPyr][Sn(AlBr_4_)_3_] (**4**) (Sn^2+^ coordination polyhedra: grey, [AlBr_4_]^−^: orange, Al_2_Br_6_: green, [EMIm]^+^/[BMPyr]^+^: grey with both possible positions displayed in (c) and (d)).

[Sn_3_(AlBr_4_)_6_](Al_2_Br_6_) (**1**), Sn(AlBr_4_)_2_ (**2**), [EMIm][Sn(AlBr_4_)_3_] (**3**) and [BMPyr][Sn(AlBr_4_)_3_] (**4**) were prepared by reacting SnCl_2_ or SnBr_2_ in a Lewis‐acidic ionic liquid established by a mixture of [Cation]Cl/Br ([Cation]: [BMIm]: 1‐butyl‐3‐methylimidazolium, [EMIm]: 1‐ethyl‐3‐methylimidazolium, [BMPyr]: 1‐butyl‐1‐methyl‐pyrrolidinium) and AlBr_3_ with a ratio of 1 : 3 (Table S1, Supporting Information). The ratio of 1 : 3 turned out to be crucial for all reactions. On the one hand, the yield drops significantly when using less AlBr_3_, until no solid compound was obtained at all. With an increasing amount of AlBr_3_, on the other hand, some AlBr_3_ remains undissolved, resulting in mixtures of the title compound and AlBr_3_. All reactions were performed in sealed, argon‐filled glass ampoules by heating to 45–100 °C and result in colorless crystals of the title compounds (Figure 1). While, in principle, all compounds can be obtained at 45 °C, crystal size and quality especially of **2**–**4** were improved by performing the reaction at 100 °C. Temperatures >150 °C lead to a decomposition of the ionic liquid as indicated by their reddish‐brown color after the reaction. In sum, the formation of the title compounds can be rationalized with the following reactions [Eqs. (1–4]:

(1) $$3\;\mathrm{SnBr}{}_{2}+8\;\mathrm{AlBr}{}_{3}\to \left[\mathrm{Sn}{}_{3}\left(\mathrm{AlBr}{}_{4}\right){}_{6}\right]\left(\mathrm{Al}{}_{2}\mathrm{Br}{}_{6}\right)$$

(2) $$\mathrm{SnCl}{}_{2}+3\;\mathrm{AlBr}{}_{3}+\left[\mathrm{BMIm}\right]\mathrm{Cl}\to \mathrm{Sn}\left(\mathrm{AlBr}{}_{4}\right){}_{2}+\left[\mathrm{BMIm}\right]{}^{+}+\left[\mathrm{AlCl}{}_{3}\mathrm{Br}\right]{}^{-}$$

(3) $$\mathrm{SnBr}{}_{2}+4\;\mathrm{AlBr}{}_{3}+\left[\mathrm{EMIm}\right]\mathrm{Cl}\to \left[\mathrm{EMIm}\right]\left[\mathrm{Sn}\left(\mathrm{AlBr}{}_{4}\right){}_{3}\right]+\mathrm{AlBr}{}_{2}\mathrm{Cl}$$

(4) $$\mathrm{SnBr}{}_{2}+3\;\mathrm{AlBr}{}_{3}+\left[\mathrm{BMPyr}\right]\mathrm{Br}\to \left[\mathrm{BMPyr}\right]\left[\mathrm{Sn}\left(\mathrm{AlBr}{}_{4}\right){}_{3}\right]$$

### Structure and composition

**Figure 2:**
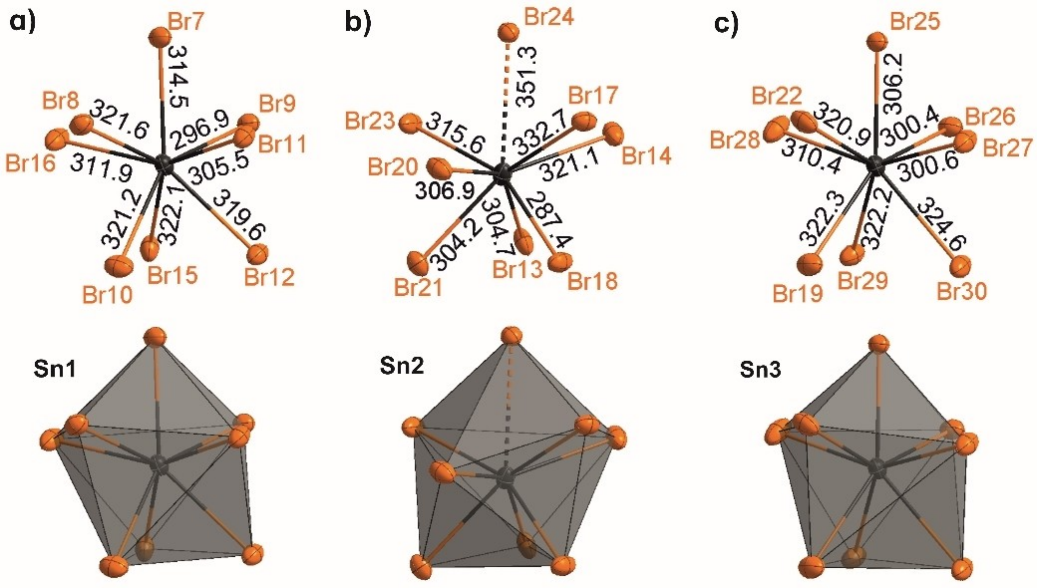
Sn^2+^ coordination and Sn−Br distances (in pm) in [Sn_3_(AlBr_4_)_6_](Al_2_Br_6_) (**1**) for the different crystallographic sites: a) Sn1, b) Sn2, c) Sn3.

[Sn_3_(AlBr_4_)_6_](Al_2_Br_6_) (**1**) crystallizes in the form of colorless bars in the monoclinic space group *P*2_1_ */c* (Table S2, Supporting Information). **1** exhibits a unique inorganic _∞_^3^[Sn_3_(AlBr_4_)_6_] network with intercalated Al_2_Br_6_ molecules (Figures 1 and Figure S1). To the best of our knowledge, such Al_2_Br_6_ intercalation is here observed for the first time. The non‐charged _∞_^3^[Sn_3_(AlBr_4_)_6_] network consists of Sn atoms that are coordinated and linked by AlBr_4_ tetrahedra, leading to an eightfold coordination of bromine around Sn^2+^ (Figure 2 and Figures S1–S4). In detail, three different Sn^2+^ sites occur, whereof Sn1 and Sn3 are largely similar to each other. Thus, Sn1 and Sn3 exhibit Sn−Br distances of 287.4(1) (Sn2−Br18) to 322.3(1) pm (Sn3−Br19) (Figure 2 and Figures S3,S4), which is comparable to SnBr_2_.^[7]^ The SnBr_8_ polyhedra can be described as significantly distorted bicapped trigonal prisms. In sum, Sn1 and Sn3 are each coordinated by four μ_2_‐coordinating AlBr_4_ tetrahedra (Figure 2, and Figure S2). In contrast, Sn2 shows (7+1) coordination, with six Sn−Br distances between 300.4(1) (Sn3−Br26) and 321.1(1) pm (Sn3−Br14) and one slightly elongated distance of 332.7(1) pm (Sn2−Br17). Finally, one Sn−Br distance is much more elongated with 351.3(1) pm (Sn2−Br24) (Figure 2), which even exceeds the longest Sn−Br distances in SnBr_2_ (338.7 pm in *α*‐SnBr_2_, 341.8 pm in *o*‐SnBr_2_).^[7b]^ This elongation can be rationalized by the (7+1) coordination of Sn^2+^ in **1** compared to the sixfold coordination of Sn^2+^ in SnBr_2_.^[7]^ In contrast to Sn1 and Sn3, Sn2 is coordinated by five AlBr_4_ tetrahedra. Three AlBr_4_ tetrahedra are μ_2_‐coordinating and two only with one bromine atom (Figure S2). All AlBr_4_ tetrahedra show distorted tetrahedral geometries with Al−Br distances of 226.4(2) (Al7−Br24) and 234.0(2) pm (Al5−Br18) as well as Br−Al−Br angles of 104.1(1) to 115.8(1)°, which fits well with literature data.[^3^, ^8^] In sum, the AlBr_4_ tetrahedra connect Sn^2+^ to an inorganic 3D network with pores in the *b,c* plane. *Such intercalation of aluminium halides – to the best of our knowledge – is observed for the first time. Moreover, such 3D networks were rarely observed for halogenidoaluminates. Thus, (AlBr_4_)‐interconnected arrangements were yet described only for Sn(C_7_H_8_)(AlBr_4_)_2_⋅C_7_H_8_, Sn(C_9_H_12_)(AlBr_4_)_2_, and (C_9_H_12_)SnBr(AlBr_4_)*.^[3]^

The pores in the _∞_^3^[Sn_3_(AlBr_4_)_6_] network are filled with non‐charged Al_2_Br_6_ molecules with similar molecular features as Al_2_Br_6_ molecules in solid or gaseous AlBr_3_.^[9]^ The Al−Br distances are 221.1(2)–221.8(2) pm for terminal Br atoms and 240.4(2)–241.9(2) pm for bridging Br atoms, which are well comparable with solid AlBr_3_ (221.9–222.3 and 240.4–242.2 pm)^[9a]^ as well as gaseous AlBr_3_ (222.7 and 242.1 pm).^[9b]^ The similar distances and angles of the Al_2_Br_6_ molecules in _∞_^3^[Sn_3_(AlBr_4_)_6_] and in solid/gaseous AlBr_3_ point to neglectable binding of the molecules with the _∞_^3^[Sn_3_(AlBr_4_)_6_] matrix. The shortest non‐bonding Al…Br contact in **1** (414 pm) is even longer than that in solid Al_2_Br_6_ (394 pm).^[10]^

**Figure 3:**
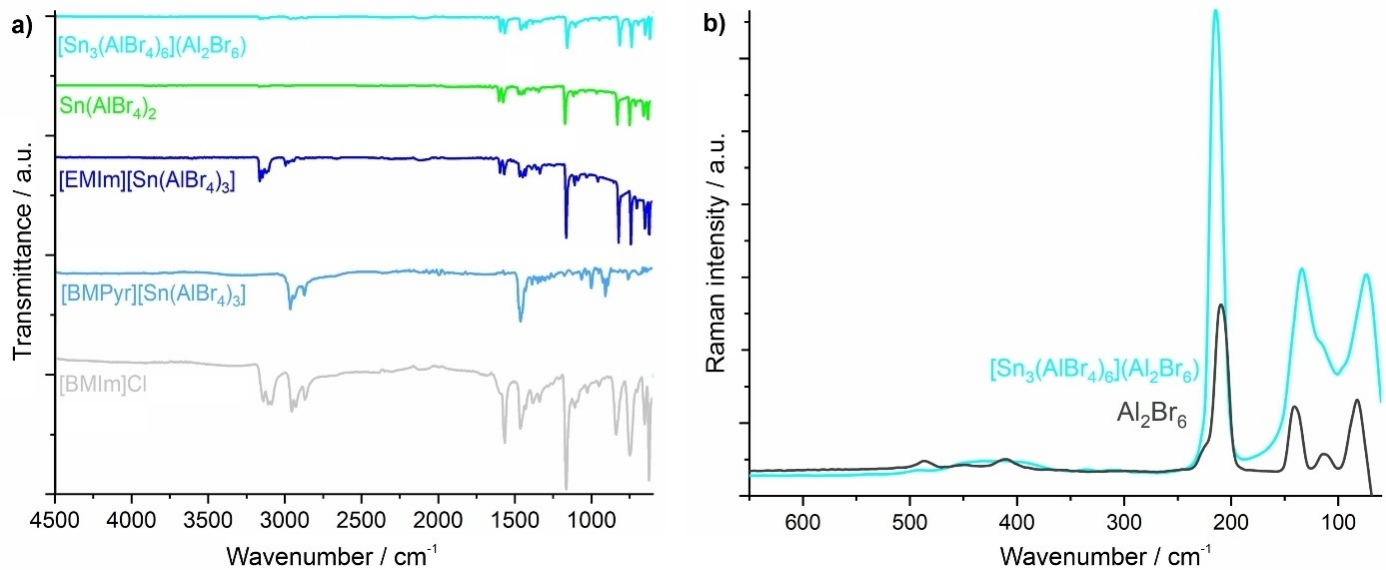
Vibrational spectra of the title compounds: a) FTIR spectra of all title compounds with [BMIm]Cl as a reference, b) Raman spectra (*λ_exc_*:532 nm) of [Sn_3_(AlBr_4_)_6_](Al_2_Br_6_) (**1**, light blue) and Al_2_Br_6_ molecules in solid AlBr_3_ (black) as a reference.

The similarity of Al_2_Br_6_ molecules in **1** with pure AlBr_3_ is further validated by Fourier‐transform infrared (FTIR) spectroscopy and Raman spectroscopy (Figure 3). In this regard, FTIR spectroscopy was performed for all title compounds. For **3** and **4**, the spectra are dominated by vibrations of the [EMIm]^+^/[BMPyr]^+^ cation (3250–2900, 3100–2800 cm^−1^). For **1** and **2**, weak vibrations of [BMIm]^+^ are also observed due to adhered ionic liquid on the crystal surfaces, which is in accordance with elemental analysis (Figure 3a). Regarding the Al_2_Br_6_ molecule in **1**, moreover, Raman spectra were recorded and exhibit vibrations similar to pure AlBr_3_ (Figure 3b),^[11]^ which again points to the single‐molecule‐like behavior of Al_2_Br_6_ in _∞_^3^[Sn_3_(AlBr_4_)_6_].^[12]^ The signals at 413, 215, and 134 cm^−1^ are attributed to slightly disturbed (<7 cm^−1^) fully symmetric terminal and bridging Al−Br stretching vibrations (representation *a_g_* of a molecule of *D*_2*h*_ symmetry) as well as to the rocking mode of the terminal AlBr_2_ group. The band at 215 cm^−1^ is superimposed by the symmetric vibration of AlBr_4_.^[13]^

**Figure 4:**
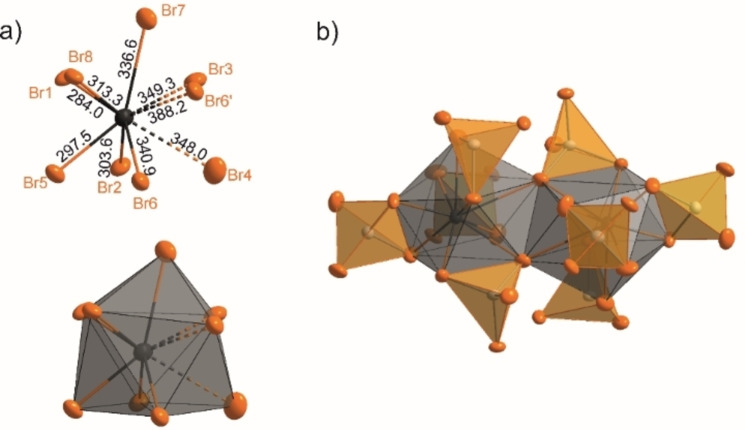
Coordination of Sn^2+^ and Sn−Br distances (in pm) in Sn(AlBr_4_)_2_ (**2**): a) polyhedra and distances, b) edge‐sharing SnBr_9_ polyhedra.

Sn(AlBr_4_)_2_ (**2**) crystallizes as colorless blocks in the space group *Pbca* (Table S2 and Figure S3 in the Supporting Information). Similar to **1**, **2** is also a 3D network with its structure isotypic to Pb(AlCl_4_)_2_ ^[14]^ and α‐Sr[GaCl_4_]_2_,^[14]^ respectively. Each Sn^2+^ ion in **2** is coordinated by nine Br atoms of four μ_2_‐coordinating AlBr_4_ tetrahedra and one Br atom of a fifth AlBr_4_ tetrahedron (Figure 4 and Figure S4). The coordination polyhedron of Sn^2+^ can either be described as a significantly distorted tricapped trigonal prism or as a capped square antiprism. In parallel to the crystallographic *c* axis, pairs of these SnBr_9_ polyhedra are found to share only one edge. There are three sets of Sn−Br distances (Figure 4). The first set ranges from 284.0(1) (Sn−Br1) to 313.3(1) (Sn−Br8) pm and compares to the shorter distances in **1** or SnBr_2_.^[7]^ The second set of 336.6(1) (Sn−Br7) to 349.3(1) pm (Sn−Br3) is in the range of the longest distances in **1** or SnBr_2_ (Table 1).^[7]^ With 388.2(1) pm (Sn−Br6), the longest distance is shorter than the sum of the van der Waals radii (403 pm)^[15]^ but longer than distances usually considered for significant interactions. These long distances are observed between Sn^2+^ and Br atoms of the two edge‐sharing SnBr_9_ polyhedra (Figure 4). The increased Sn−Br distances in **2**, compared to **1**, can be ascribed to the higher coordination number of nine in **2**. However, by analogy with the isotypic structures Pb(AlCl_4_)_2_ ^[12]^ and α‐Sr[GaCl_4_]_2_,^[12b]^ the description as SnBr_9_ polyhedra seems reasonable. In accordance with **1**, all AlBr_4_ tetrahedra in **2** are also slightly distorted with Al−Br distances and angles similar to literature data.[^3^, ^8^]

**Table 1:** Sn−Br distances in [Sn_3_(AlBr_4_)_6_](Al_2_Br_6_) (**1**), Sn(AlBr_4_)_2_ (**2**), [EMIm][Sn(AlBr_4_)_3_] (**3**) and [BMPyr][Sn(AlBr_4_)_3_] (**4**) in comparison to selected reference compounds.

Compound	Sn^2+^ coordination	Short distances [pm]	Long distances [pm]
**1**	8	287.4(1)–322.3(1)	/
	7+1	300.4(1)–332.7(1)	351.3(1)
**2**	4+4+1	284.0(1)–313.3(1)	336.6(1)–349.3(1), 388.2(1)
**3**	8	300.2(1)–322.1(1)	/
**4**	8	297.3(1)–321.6(1)	/
α‐SnBr_2_ ^[7b]^	6	285.9–333.7	/
*o*‐SnBr_2_ ^[7b]^	6	280.6–312.2	341.8
Sn(C_9_H_12_)(AlBr_4_)_2_^*[8a]*^	6+η^6^‐C_9_H_12_	291.5–338.0	/

Finally, [EMIm][Sn(AlBr_4_)_3_] (**3**) and [BMPyr][Sn(AlBr_4_)_3_] (**4**) crystallize as colorless, elongated plates in the monoclinic space group *C*2*/c* (Table S2). As most interesting building unit, both contain infinite _∞_^1^[Sn(AlBr_4_)_3_]^−^ chains (Figure S5), similar to those previously observed in, for example, [BMIm][Sn(AlCl_4_)_3_].[^6^, ^16^] In the _∞_^1^[Sn(AlBr_4_)_3_]^−^ chains, each Sn^2+^ is surrounded by three μ_2_‐coordinating AlBr_4_ tetrahedra and two μ_1_‐coordinating Br^−^ ions of two additional AlBr_4_ tetrahedra resulting in an eightfold coordination of Sn^2+^ with Br in a distorted squared anti‐prismatic geometry (Figure 1 and Figures S3,S4). Four of the five AlBr_4_ tetrahedra interlink neighboring Sn^2+^ in a μ_2_‐μ_1_ fashion to chains along the crystallographic *c* axis (Figure S5). The residual fifth AlBr_4_ tetrahedron coordinates two Br to one Sn atom, together resulting in an eightfold coordination of Sn^2+^. Such coordination was previously observed in, for example, Sn(C_9_H_12_)(AlBr_4_)_2_.^[8a]^

For **3**, the Sn−Br distances are 300.2(1) pm (Sn−Br5) to non‐bridging AlBr_4_, 313.4(1) and 322.1(1) pm (Sn−Br1, Sn−Br2) to μ_2_‐coordinating AlBr_4_, and 321.0(1) pm (Sn−Br3) to corner‐sharing AlBr_4_. For **4**, the Sn−Br distances are 297.3(1) pm (Sn−Br5) to the non‐bridging AlBr_4_, 312.8(1) and 321.6(1) pm (Sn−Br1, Sn−Br2) to the μ_2_‐coordinating AlBr_4_ and 320.8(1) pm (Sn−Br3) to the corner‐sharing AlBr_4_ (Table 1 and Figure S4). These distances are similar to the observed distances in **1** and **2** and agree with literature data, for example, 291.4–338.0 pm in Sn(C_9_H_12_)(AlBr_4_)_2_.^[8a]^ Again, all AlBr_4_ tetrahedra in **3** and **4** are slightly distorted with the expected distances and angles.[^3^, ^8^] Moreover, distances and angles of the [EMIm]^+^ and [PMPyr]^+^ cations are as expected. For both, two more‐or‐less mirrored positions were observed in the crystal structure, which were tackled by split atom positions for all atoms with 50 % probability of finding for each position.

### Material properties

To validate of the chemical composition of the title compounds, energy‐dispersive X‐ray spectroscopy (EDXS), elemental analysis (C,H,N), and thermogravimetry (TG) were performed. First of all, EDXS confirms the presence of Sn and Al/Br. Here, it must be noticed that Al and Br cannot be reliably distinguished due to the similar energy of the Al−*K*_*α*_ emission (1.486 eV) and the Br−*L*_*α*_ emission (1.480 eV). Moreover, single crystals of the title compounds decompose rapidly under electron bombardment in vacuum (30 kV) due to Al_2_Br_6_ release (Figure S6, Supporting Information). Elemental analysis shows the absence of C/H/N for **1** and **2** and confirms the presence of [EMIm]^+^ and [BMPyr]^+^ in **3** and **4** (Table S3). Due to total decomposition of the title compounds, finally, TG allows to quantify the chemical composition and to study the thermal properties. All title compounds show a one‐step decomposition with dark grey residues of elemental tin remaining (Figure S7). Decomposition of **1** starts at 210 °C with a total weight loss of 85.8 %, whereas **2** decomposes at 270 °C with a total loss of 90.6 %, which can be ascribed to the following reactions [Eqs. (5,6]:

(5) $$\left[\mathrm{Sn}{}_{3}\left(\mathrm{AlBr}{}_{4}\right){}_{6}\right]\left(\mathrm{Al}{}_{2}\mathrm{Br}{}_{6}\right)\to 3\;\mathrm{Sn}↓\left(12.0\;\%\right)+4\;\mathrm{Al}{}_{2}\mathrm{Br}{}_{6}↑\left(71.9\;\%\right)+3\;\mathrm{Br}{}_{2}↑\left(16.1\;\%\right)$$

(6) $$\mathrm{Sn}\left(\mathrm{AlBr}{}_{4}\right){}_{2}\to \mathrm{Sn}↓\left(14.6\;\%\right)+\mathrm{Al}{}_{2}\mathrm{Br}{}_{6}↑\left(65.7\;\%\right)+\mathrm{Br}{}_{2}↑\left(19.7\;\%\right)$$

**3** and **4** show comparable decomposition processes starting at 75 and 80 °C with total weight losses of 91.2 and 87.6 %, respectively [Eqs. (7,8]:

(7) $$\left[C{}_{6}H{}_{11}N{}_{2}\right]\left[\mathrm{Sn}\left(\mathrm{AlBr}{}_{4}\right){}_{3}\right]\to \mathrm{Sn}↓\left(9.3\;\%\right)+1.5\mathrm{Al}{}_{2}\mathrm{Br}{}_{6}↑\left(63.1\;\%\right)+1.5\mathrm{Br}{}_{2}↑\left(18.9\;\%\right)+C{}_{2}H{}_{4}↑\left(2.2\;\%\right)+N\left(C{}_{3}H{}_{4}\right){}_{2}\left(\mathrm{CH}{}_{3}\right)↑\left(6.5\;\%\right)$$

(8) $$\left[C{}_{9}H{}_{20}N\right]\left[\mathrm{Sn}\left(\mathrm{AlBr}{}_{4}\right){}_{3}\right]\to \mathrm{Sn}↓\left(9.1\;\%\right)+1.5\mathrm{Al}{}_{2}\mathrm{Br}{}_{6}↑\left(61.5\;\%\right)+1.5\mathrm{Br}{}_{2}↑\left(18.5\;\%\right)+C{}_{4}H{}_{8}↑\left(4.3\;\%\right)+\mathrm{NH}\left(C{}_{4}H{}_{8}\right)\left(\mathrm{CH}{}_{3}\right)↑\left(6.6\;\%\right)$$

**Figure 5:**
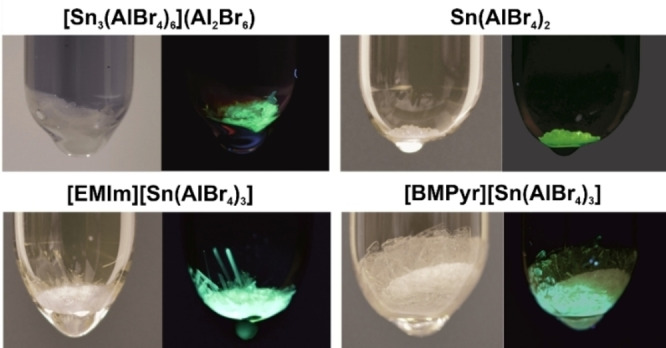
Photos of [Sn_3_(AlBr_4_)_6_](Al_2_Br_6_) (**1**), Sn(AlBr_4_)_2_ (**2**), [EMIm][Sn(AlBr_4_)_3_] (**3**) and [BMPyr][Sn(AlBr_4_)_3_] (**4**) under daylight and under UV irradiation.

Regarding the optical properties, first of all, ultraviolet‐visible (UV‐Vis) spectroscopy was performed (Figure S8). Here, weak absorption is observed below 420 (**1**), 450 (**2**), and 400 nm (**3**, **4**), which is in accordance with the colorless appearance of the title compounds (Figures 1, 5). The UV‐Vis spectra are compared to those of SnBr_2_ and AlBr_3_ as references. Valence‐band‐to‐conduction‐band absorption is observed for SnBr_2_ with high intensity below 400 nm, which is in accordance with its yellowish color.^[17]^ In contrast, the absorption of the title compounds compares to colorless AlBr_3_ (Figure S8). Here, weak absorption at 230–400 nm occurs and can be ascribed to Br^−^→Al^3+^ ligand‐to‐metal charge‐transfer (LMCT) absorption. Intense valence‐band to conduction‐band absorption is observed for AlBr_3_ below 230 nm. Similar to AlBr_3_, thus, the weak absorption at 230 to 420/450/400 nm can be attributed to the title compounds, too. The additional absorption of the title compounds, therefore, is attributed to an LMCT transition.

**Figure 6:**
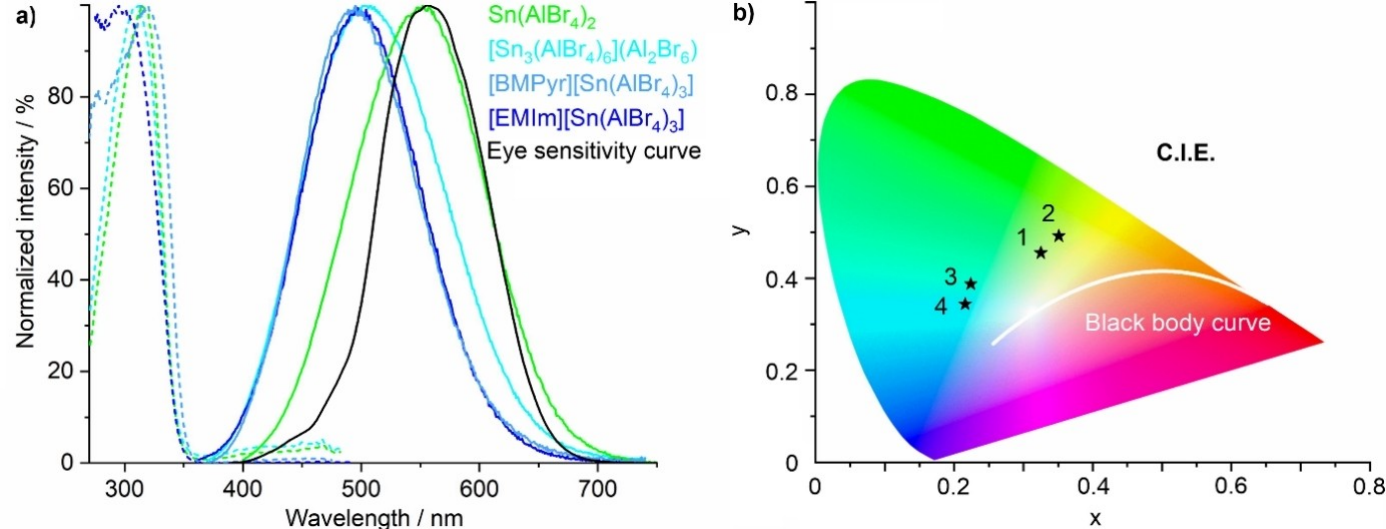
Luminescence properties of [Sn_3_(AlBr_4_)_6_](Al_2_Br_6_) (**1**), Sn(AlBr_4_)_2_ (**2**), [EMIm][Sn(AlBr_4_)_3_] (**3**) and [BMPyr][Sn(AlBr_4_)_3_] (**4**): a) photoluminescence spectra (with eye‐sensitivity curve),^[19]^ b) color coordinates of the emission of **1**–**4** in the C.I.E. diagram.

While performing UV‐Vis spectroscopy, we realized that all title compounds also show luminescence (Figure 5), which was quantified by photoluminescence spectroscopy (Figure 6a). Accordingly, **1**–**4** exhibit similar absorption below 350 nm. Upon UV excitation (280–350 nm), they show bluish to greenish white emission with emission bands at 400 to 700 nm (Figures 5, 6a). The emission can be related to a 5*s* ^1^ *p* ^1^→5*s* ^2^ *p* ^0^ transition on Sn^2+^. Most interestingly, the luminescence process is very efficient with quantum yields >50 % for all compounds (Table 2). Specifically, **3** and **4** exhibit outstanding quantum yields of 98 and 99 %. Although, Sn^2+^‐based emission is well‐known in principle, the quantum yield is limited to 80–85 % or much below due to the strong coupling of *s* and *p* orbitals with the lattice.[^1^, ^2^] Moreover, Sn^2+^ as a luminescence center is usually applied as a dopant (5–10 mol‐ %) in a non‐luminescent host lattice to avoid concentration quenching at higher concentrations (>10 mol‐ %).[^2^, ^3^] Thus, the efficient Sn^2+^‐based luminescence is surprising, and the observed quantum yields near unity, to the best of our knowledge, are the highest ever observed for Sn^2+^. The extremely high quantum yields can be explained by the rigid binding of Sn^2+^ in the bromido‐aluminate host lattices as well as by the large distance between the luminescent Sn^2+^ centers (≥673.5(1) pm), so that no concentration quenching occurs despite of molar quantities of Sn^2+^ in the lattice.

**Table 2:** Luminescence properties of [Sn_3_(AlBr_4_)_6_](Al_2_Br_6_) (**1**), Sn(AlBr_4_)_2_ (**2**), [EMIm][Sn(AlBr_4_)_3_] (**3**) and [BMPyr][Sn(AlBr_4_)_3_] (**4**).

Compound	Max. excitation [nm]	Max. emission [nm]	FWHM [nm]	CIE color coordinates x, y	Quantum yield [%]
**1**	310	509	139	0.351, 0.492	87
**2**	315	551	132	0.325, 0.455	56
**3**	295	497	117	0.224, 0.387	99
**4**	318	495	115	0.216, 0.344	98

Beside the intense luminescence and partly outstanding quantum yield, another interesting feature is the shift of the color coordinates in the C. I. E diagram (C. I. E.: Commission International de l′Eclairage)^[1]^ between the compounds **1**–**4**, which is more or less parallel to the blackbody radiation curve (Table 2, Figure 6b). Specifically, the emission of **2** is almost similar to the eye‐sensitivity curve (Figure 6a). By certain adaption of the host lattice (e. g., partial exchange of halogen, partial exchange of aluminium),^[18]^ it could be possible to shift the color coordinates to the blackbody radiation curve and to obtain highly efficient white‐light emitting phosphors.

## Conclusions

Four novel tin bromido aluminate networks were prepared by reaction of SnCl_2_/SnBr_2_ in Lewis‐acidic ionic liquids based on mixtures of [BMIm]Cl/Br or [BMPyr]Br and AlBr_3_. Specifically, these are [Sn_3_(AlBr_4_)_6_][Al_2_Br_6_] (**1**), Sn(AlBr_4_)_2_ (**2**), [EMIm][Sn(AlBr_4_)_3_] (**3**) and [BMPyr][Sn(AlBr_4_)_3_] (**4**). The crystal structures are characterized by AlBr_4_‐coordinated Sn^2+^ cations resulting in 3D networks in **1** and **2** as well as chain‐like structures in **3** and **4**. Interestingly, the _∞_^3^[Sn_3_(AlBr_4_)_6_] network of **1** contains isolated Al_2_Br_6_ molecules with distances/angles similar to Al_2_Br_6_ molecules in pure AlBr_3_. In general, such tin bromido aluminate networks as observed in the title compounds were rarely reported in the literature until now.

Surprisingly, all title compounds show intense bluish white to greenish white emission originating from Br^−^→Al^3+^ ligand‐to‐metal charge‐transfer excitation, followed by 5*s* ^2^ *p* ^0^←5*s* ^1^ *p* ^1^ emission on Sn^2+^. Especially, **3** and **4** have outstanding quantum yields of 98 and 99 %, which are the highest values observed for Sn^2+^‐based luminescence so far. The efficient photoluminescence can be explained by the rigid binding of Sn^2+^ in the bromido‐aluminate network and the great distance between the luminescent Sn^2+^ centers (≥673.5(1) pm). As a result, loss processes due to thermal quenching and concentration quenching are low. Since the color coordinates of **1**–**4** are close to the blackbody radiation curve, certain modification of the host lattice could potentially lead to efficient white‐light emitters with quantum yields near unity.

## Experimental Section

### Synthesis

*General*. All reactions and sample handling were carried out under dried argon atmosphere using standard Schlenk techniques or argon‐filled glove boxes. Reactions were performed in Schlenk flasks and glass ampoules that were evacuated (*p*<10^−3^ mbar), heated and flushed with argon thrice prior to use. The starting materials SnCl_2_ (99.99 %, Sigma Aldrich), SnBr_2_ (99 %, ABCR), and AlBr_3_ (99 %, ABCR) were used as received. [BMIm]Cl (99 %, Iolitec), [EMIm]Cl (99 %, Iolitec), and [BMPyr]Br (99 %, Iolitec) were dried under reduced pressure (10^−3^ mbar) at 130 °C for 48 h. All compounds were handled and stored in argon‐filled glove boxes (MBraun Unilab, *c*(O_2_, H_2_O)<0.1 ppm).

*[Sn_3_(AlBr_4_)_6_](Al_2_Br_6_) (**1**)*. 150 mg (0.24 equiv., 0.54 mmol) of SnBr_2_, 500 mg (1 equiv., 2.28 mmol) of [BMIm]Cl and 1.827 mg (3 equiv., 6.85 mmol) of AlBr_3_ were heated under argon in a sealed glass ampoule for 2 weeks at 45 °C. At the beginning, a yellowish ionic liquid was formed, in which crystals of **1** grow over several days as colorless cuboids or bars. Crystals can be separated from the IL by filtration through a glass filter to result in a phase‐pure compound with 20–30 % yield. **1** is highly sensitive to moisture and hydrolyses in air within seconds. To obtain large (up to 1 mm) single crystals of **1**, the reaction can be alternatively performed at 100 °C for 4 h. As a result, however, a mixture of **1** and crystals of SnBr_2_ was obtained.

*Sn(AlBr_4_)_2_ (**2**)*. 100 mg (0.18 equiv., 0.53 mmol) of SnCl_2_, 500 mg (1 equiv., 2.86 mmol) of [BMIm]Cl and 2.290 mg (3 equiv., 8.58 mmol) of AlBr_3_ were heated under argon in a sealed glass ampoule for 4 h at 100 °C. After cooling to room temperature with a rate of 1 K/h, **2** was obtained as colorless bars in a yellowish ionic liquid with 20–25 % yield. Yield and crystal quality were depending on the applied amount of ionic liquid. With less ionic liquid, the yield was increased, however, the crystal quality decreased and non‐dissolved AlBr_3_ remained in the sample. With more ionic liquid, the yield decreased even further due to the higher solubility. **2** is highly sensitive to moisture and hydrolyses in air within seconds. Hence, **2** needs to be handled under inert conditions. Similar to **1**, the crystals can be separated from the IL via filtration through a glass filter.

*[EMIm][Sn(AlBr_4_)_3_] (**3**)*. 100 mg (0.18 equiv., 0.36 mmol) of SnBr_2_, 300 mg (1 equiv., 2.05 mmol) of [EMIm]Cl and 1.637 mg (3 equiv, 6.14 mmol) of AlBr_3_ were heated under argon in a sealed glass ampoule for 4 h at 100 °C. After cooling to room temperature with a rate of 1 K/h, **3** was obtained as large colorless plates in a yellowish ionic liquid with a yield of 25–30 %. Again, yield and crystal quality were depending on the applied concentrations. **3** is highly sensitive to moisture and hydrolyses in air within seconds. Hence, **3** needs to be handled under inert conditions. Crystals can be separated from the IL via filtration through a glass filter.

*[BMPyr][Sn(AlBr_4_)_3_] (**4**)*. 150 mg (0.24 equiv., 0.54 mmol) of SnBr_2_, 507 mg (1 equiv., 2.28 mmol) of [BMPyr]Br and 1.826 mg (3 equiv., 6.85 mmol) of AlBr_3_ were heated under argon in a sealed glass ampoule for 4 h at 100 °C. After cooling to room temperature with a rate of 1 K/h, **4** was obtained phase pure as colorless needles in a yellowish ionic liquid with a yield of 35–40 %. **4** is extremely sensitive to moisture and needs to be handled under inert conditions. The crystals can be separated from the IL via filtration through a glass filter.

### Analytical equipment

*Single‐crystal structure analysis*. For single crystal structure analysis, suitable crystals of **1**–**4** were manually selected, covered by inert‐oil (perfluoropolyalkylether), placed on a microgripper (MiTeGen), and, thereafter, immediately frozen, which prevented any contact between the crystals and air/moisture. Data collection for **2**–**4** was performed at 200 or 213 K on an IPDS II image‐plate diffractometer (Stoe, Darmstadt) using Mo‐K_α_ radiation (λ=0.71073 Å, graphite monochromator). Data collection for **1** was performed at 180 K on a Stoe StadiVari Diffractometer with Euler geometry (Stoe, Darmstadt) using Ga‐K_α_ radiation (λ=1.34143 Å, graded multilayer mirror as monochromator). Data reduction and multiscan absorption correction were conducted with the X‐AREA software package and STOE LANA (version 1.75).^[20]^ Space group determination based on systematic absences of reflections was performed by XPREP. Using Olex2,^[21]^ the structure was solved with the SHELXT^[22]^ structure solution program using Intrinsic Phasing and refined with the SHELXL refinement package using Least Squares minimization. All non‐hydrogen atoms were refined anisotropically. Hydrogen atoms were constructed geometrically. Detailed information on crystal data and structure refinements are listed in the Supporting Information (Table S2). DIAMOND was used for all illustrations.^[23]^ Refinement was checked with PLATON.^[24]^

Deposition Numbers 2202778 (for [BMPyr][Sn(AlBr_4_)_3_]), 2202779 (Sn(AlBr_4_)_2_), 2202780 (for [Sn_3_(AlBr_4_)_6_](Al_2_Br_6_)), 2202781 (for [EMIm][Sn(AlBr_4_)_3_]) contain the supplementary crystallographic data for this paper. These data are provided free of charge by the joint Cambridge Crystallographic Data Centre and Fachinformationszentrum Karlsruhe Access Structures service.

*Photoluminescence spectroscopy*. Excitation and emission spectra were recorded using a photoluminescence spectrometer Horiba Jobin Yvon Spex Fluorolog 3, equipped with a 450 W Xenon lamp, double monochromators for excitation and emission, an integrating sphere (Ulbricht sphere), and a photomultiplier as detector. The determination of the quantum yield was performed according to Friend et al.^[25]^ First of all, the diffuse reflection of the samples was determined under excitation conditions. Thereafter, the emission was measured at this excitation wavelength. Integration over the reflected and emitted photons by use of the Ulbricht sphere resulted in the absolute quantum yield. Corrections were made regarding the spectral power of the excitation source, the reflection behavior of the Ulbricht sphere, and the sensitivity of the detector. The samples were placed inside a glove box in Ar‐filled air‐tight cuvettes, transferred to the spectrometer and then measured.

### Supporting Information Summary

Data related to additional analytical methods (EDXS, EA, TG, FTIR, Raman, UV‐Vis) are deposited in the Supporting Information.

## Conflict of interest

The authors declare no conflict of interest.

1

## Supporting information

As a service to our authors and readers, this journal provides supporting information supplied by the authors. Such materials are peer reviewed and may be re‐organized for online delivery, but are not copy‐edited or typeset. Technical support issues arising from supporting information (other than missing files) should be addressed to the authors.

Supporting Information

## Data Availability

The data that support the findings of this study are available from the corresponding author upon reasonable request.
