# Supplementary material for: Tin Bromido Aluminate Networks with Bright Luminescence
Source: ChemistryOpen. 2023 Feb 22;12(2):e202200226. doi: 10.1002/open.202200226 (PMC9944849; doi:10.1002/open.202200226)
Supplement: Supplementary file 1 — Supporting Information [file OPEN-12-e202200226-s001.pdf]

ChemistryOpen

Supporting Information

Tin Bromido Aluminate Networks with Bright Luminescence

Silke Wolf, Ralf Köppe, Peter W. Roesky, and Claus Feldmann*

Content

1. Synthesis and Analytical Techniques

2. Structural Properties

3. Chemical Composition

4. Material Properties

1. Synthesis and Analytical Techniques

$[Sn_3(AlBr_4)_6](Al_2Br_6)$ (**1**), $Sn(AlBr_4)_2$ (**2**), $[EMIm][Sn(AlBr_4)_3]$ (**3**) and $[BMPyr][Sn(AlBr_4)_3]$ (**4**) were prepared by reacting $SnCl_2$ or $SnBr_2$ in a Lewis-acidic ionic liquid established by a mixture of [Cation]Cl/Br ([Cation]: [BMIm]: 1-butyl-3-methylimidazolium, [EMIm]: 1-ethyl-3-methylimidazolium, [BMPyr]: 1-butyl-1-methyl-pyrrolidinium) and $AlBr_3$ with the experimental conditions summarized in Table S1 (main manuscript: reaction equations (1-4)) .

Table S1. Experimental conditions to obtain $[Sn_3(AlBr_4)_6](Al_2Br_6)$ (**1**), $Sn(AlBr_4)_2$ (**2**), $[EMIm][Sn(AlBr_4)_3]$ (**3**) and $[BMPyr][Sn(AlBr_4)_3]$ (**4**).

**Compound**	**Starting material**	**Ionic liquid**	**Temperature /°C**	**Time /h**
**1**	$SnBr_2$	$[BMIm]Br + 3\ AlBr_3$	45	2 weeks
**1 + $SnBr_2$**	$SnBr_2$	$[BMIm]Br + 3\ AlBr_3$	100	4
**2**	$SnCl_2$	$[BMIm]Cl + 3\ AlBr_3$	100	4
**3**	$SnBr_2$	$[EMIm]Cl + 3\ AlBr_3$	100	4
**4**	$SnBr_2$	$[BMPyr]Br + 3\ AlBr_3$	100	4

Energy dispersive X-ray spectroscopy (EDXS) was performed using an Ametec EDAX mounted on a Zeiss SEM Supra 35 VP scanning electron microscope at 30 kV electron energy. Sample were prepared in a glove-box by selecting single crystals under a microscope that were fixed on a conductive carbon pad on an aluminum sample holder. The samples were handled under inert conditions during transport in an airtight transfer module and sample preparation.

Elemental analysis (C/H/N/S analysis) was performed via thermal combustion with an Elementar Vario Microcube device (Elementar, Germany) at a temperature of 1,100 °C. The samples were prepared in the glove-box and transferred to the elemental analysis in air-tight containers. The samples were either combusted in tin or silver capsules, which are both temporarily air-tight.

Thermogravimetry (TG) was carried out with a Netzsch STA 449 F3 Jupiter device using α-Al_2O_3 as crucible material and reference. Buoyancy effects were corrected by baseline subtraction of a blank measurement. The samples were measured under dried nitrogen up to 800 °C with a heating rate of 10 K/min. The samples were prepared by filtration through a glass filter under argon. Inside an Ar-filled glovebox, the sample was transferred to an α-Al_2O_3 crucible with a lid. Afterwards the crucible was transferred to the thermogravimetry in an air-tight container and placed inside the device with

N_2 flux. Immediately thereafter, the whole measurement chamber was evacuated and refilled with dried nitrogen before staring the measurement.

Fourier-transform infrared (FT-IR) spectra were recorded on a Bruker Vertex 70 FT-IR spectrometer (Bruker). The samples were measured as pellets in KBr. Thus, 300 mg of dried KBr and 0.5-1.0 mg of the title compound were carefully pestled together and pressed to a thin pellet inside a glovebox. These pellets were placed in the sample holder of the device inside an Ar-filled glovebox and transferred in an air-tight container into the spectrometer. The sample holder was placed inside the already N_2-filled measurement chamber with nitrogen flux.

Raman spectroscopy. Raman spectra were recorded on a Bruker Raman microscope Senterra II using its 532 nm laser. The combination of the CCD detector and the grating (400 lines/mm) leads to a resolution of 4 cm^{-1}. The samples consisted of selected single crystals, which were cleaned from the ionic liquid by immersing in perfluorinated polyether and crushing into smaller pieces. Thereafter, these samples were fixed inside an Ar-filled glovebox on the inner side of an Ar-flushed and preheated glass tube on a small area of approximately 1 mm^2 in size and measured within the closed glass tube. The power of the laser was set to 25 mW ($Sn_3(AlBr_4)_6](Al_2Br_6)$ (**1**)) or 6 mW (Al_2Br_6), respectively.

Optical spectroscopy (UV-Vis) of powder samples was recorded on a Shimadzu UV-2700 spectrometer, equipped with an integrating sphere, in a wavelength interval of 250-800 nm against $BaSO_4$ as reference. 10 mg of sample were pestled together with dried $BaSO_4$ and filled into an air-tight sample holder inside an Ar-filled glovebox. Afterwards, the Ar-filled sample holder was transferred to the spectrometer for measurement.

2. Structural Properties

For single crystal structure analysis, suitable crystals were manually selected, covered by inert-oil (perfluoropolyalkylether), and placed on a micro gripper (MiTeGen). Data collection for **2-4** was performed at 200 or 213 K on an IPDS II image-plate diffractometer (Stoe, Darmstadt) using Mo-K_α radiation (λ = 0.71073 Å, graphite monochromator). Data collection for **1** was performed at 180 K on a Stoe StadiVari Diffractometer with Euler geometry (Stoe, Darmstadt) using Ga-K_α radiation (λ = 1.34143 Å, graded multilayer mirror as monochromator). Detailed information on crystal data and structure refinements are listed in Table S2.

Table S2. Crystallographic and refinement details of $[Sn_3(AlBr_4)_6](Al_2Br_6)$ (**1**), $Sn(AlBr_4)_2$ (**2**), $[EMIm][Sn(AlBr_4)_3]$ (**3**) and $[BMPyr][Sn(AlBr_4)_3]$ (**4**).

**Data**	**1**	**2**	**3**	**4**
**Sum formula**	$Br_{30}Al_8Sn_3$	Br_8Al_2Sn	$C_6H_{11}N_2Br_{12}Al_3Sn$	$C_9H_{20}NBr_{12}Al_3Sn$
Crystal system	Monoclinic	Orthorhombic	Monoclinic	Monoclinic
Space group	*P*2₁/*c*	*Pbca*	*C2/c*	*C2/c*
Lattice parameters	a = 2171.1(1) pm<br>b = 1926.7(1) pm<br>c = 1432.9(1) pm<br>β = 106.0(1)°	a = 1277.6(1) pm<br>b = 1095.3(1) pm<br>c = 2114.4(3)	a = 1588.9(2) pm<br>b = 1480.1(3) pm<br>c = 1249.5(3) pm<br>β = 97.3(1)°	a = 1556.8(1) pm<br>b = 1596.7(1) pm<br>c = 1256.0(1) pm<br>β = 97.2(1)°
Cell volume, V	5760.7×10^6 pm^3	2958.7×10^6 pm^3	2914.5×10^6 pm^3	3097.6×10^6 pm^3
Formula units per cell, Z	4	8	4	4
Calculated density, ρ	3.424 g cm^{-3}	3.646 g cm^{-3}	2.894 g cm^{-3}	2.789 g cm^{-3}
Measurement limits	$-28 \leq h \leq 28$,<br>$-22 \leq k \leq 25$,<br>$-5 \leq l \leq 18$	$-15 \leq h \leq 15$,<br>$-13 \leq k \leq 11$,<br>$-19 \leq l \leq 26$	$-21 \leq h < 21$;<br>$-20 \leq k < 19$,<br>$-16 \leq l < 17$	$-21 \leq h \leq 21$,<br>$-21 \leq k \leq 18$,<br>$-17 \leq l \leq 13$
2 Theta range for data collection	5.4 to 125.0°	3.8 to 58.6°	3.8 to 58.6°	3.7 to 58.4°
Wavelength	Ga-K_α<br>(λ = 1.34143Å)	Mo-K_α<br>(λ = 0.71073 Å)	Mo-K_α<br>(λ = 0.71073 Å)	Mo-K_α<br>(λ = 0.71073 Å)
Linear absorption coefficient, µ	24.685 mm^{-1}	23.407 mm^{-1}	17.418 mm^{-1}	16.391 mm^{-1}
Number of reflections	35198<br>(13384 independent)	10256<br>(2899 independent)	11494<br>(3844 independent)	11032<br>(4171 independent)
Refinement method	Full-matrix least-squares on F^2	Full-matrix least-squares on F^2	Full-matrix least-squares on F^2	Full-matrix least-squares on F^2
Merging, R_{int}	0.028	0.046	0.053	0.083
Number of parameters	371	100	146	164
Residual electron density ($e^- \times 10^{-6}$ pm$^{-3)}$	1.67 to -1.61	1.57 to -1.19	1.14 to -1.57	1.70 to -1.54
Figures of merit				
R1 ($I \geq 4\sigma_I$)	0.032	0.033	0.040	0.048
R1 (all)	0.045	0.060	0.072	0.065
wR2 (all data)	0.073	0.074	0.130	0.122
GooF	0.955	0.833	0.942	0.984

To illustrate the 3D network structure of **1**, different views of the unit cell are shown (Figure S1). Moreover, the coordination of Sn^{2+} with $[AlBr_4]^-$ and the resulting connectivity for the different Sn^{2+} sites of **1** are shown (Figure S2). To illustrate the connectivity in **1** and **2**, unit cells in a wire-and-sticks model are shown (Figure S3). Here, Sn–Br distances above the longest distance in $SnBr_2$ are illustrated as dotted lines. To compare the coordination of Sn^{2+} and Sn–Br distances, coordination polyhedra and the respective distances are shown for all title compounds (Figure S4). Finally, the almost identical ${}_\infty^1[Sn(AlBr_4)_3]^{n-}$ chains in $[EMIm][Sn(AlBr_4)_3]$ (**3**) and $[BMPyr][Sn(AlBr_4)_3]$ (**4**) are illustrated (Figure S5).

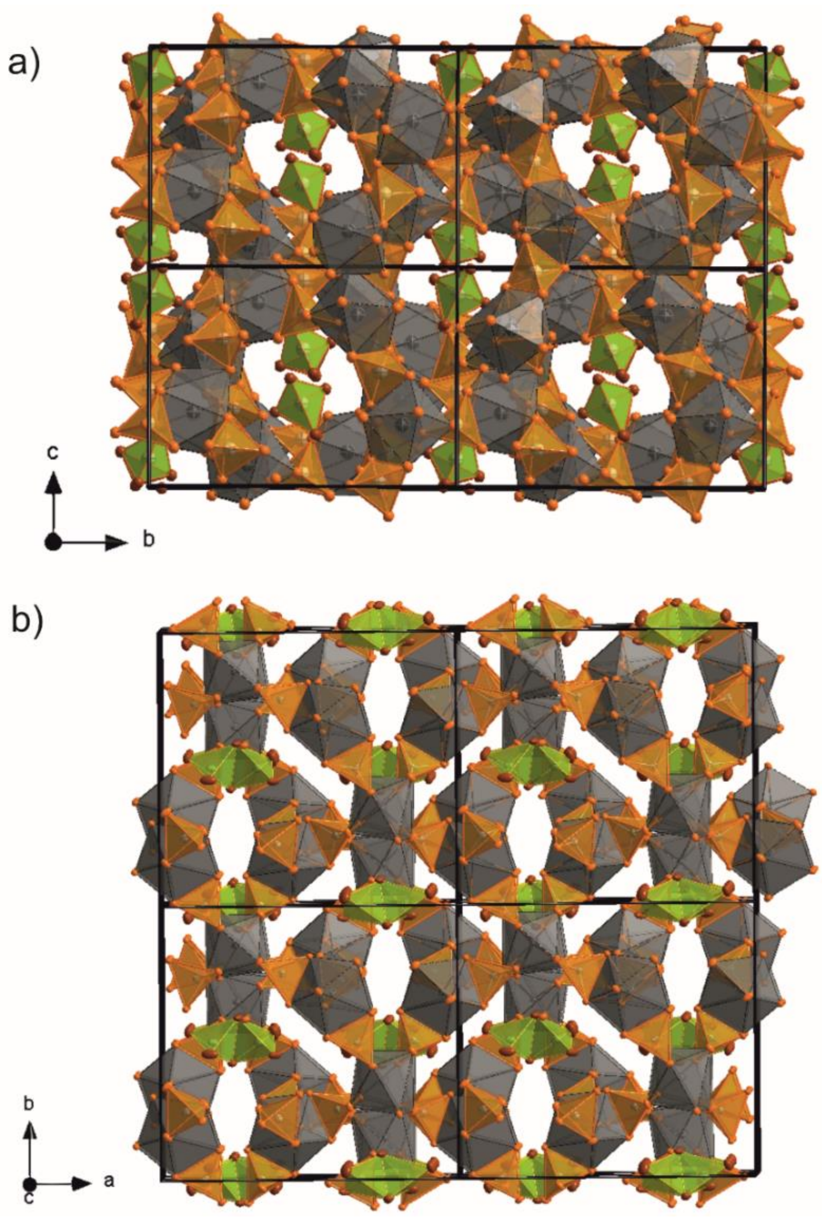

Figure S1. (2×2×2) Super cells of $[Sn_3(AlBr_4)_6](Al_2Br_6)$ (**1**) to illustrate the porous network with encapsulated Al_2Br_6 molecules: a) view of the *b*,*c* plane; view of the *a*,*b* plane (Sn^{2+} polyhedra: grey, $[AlBr_4]^-$ tetrahedra: orange, Al_2Br_6: green).

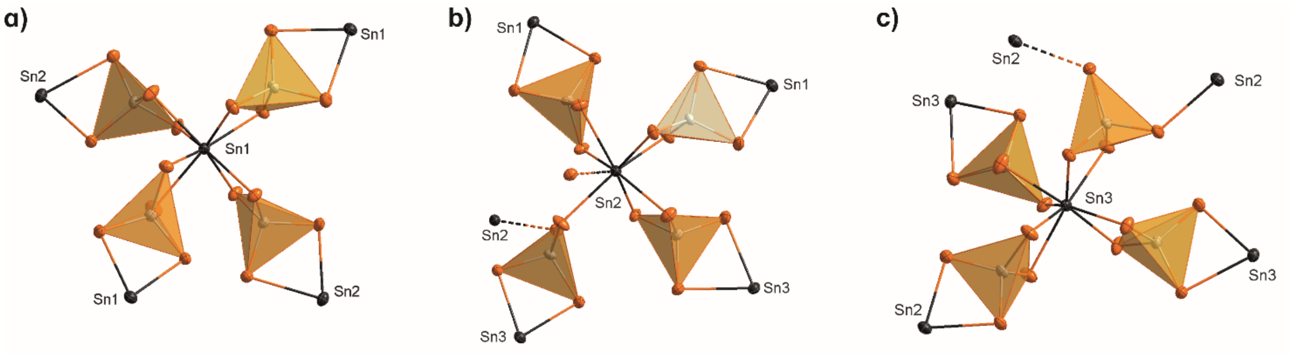

Figure S2. Coordination of Sn^{2+} with $[AlBr_4]^-$ in $[Sn_3(AlBr_4)_6](Al_2Br_6)$ (**1**): a) Sn1, b) Sn2, c) Sn3**.**

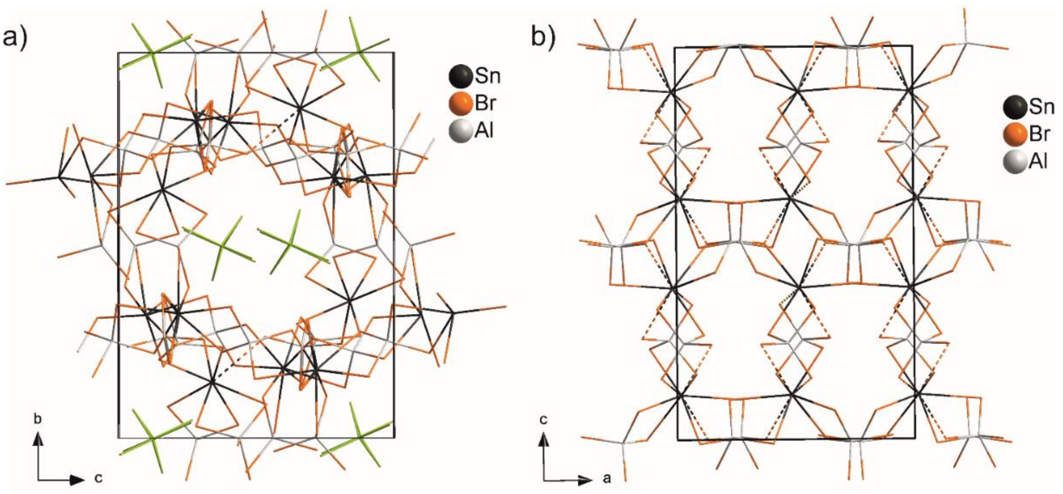

Figure S3. Unit cells of a) $[Sn_3(AlBr_4)_6](Al_2Br_6)$ (**1**) with Al_2Br_6 molecules in green and b) $Sn(AlBr_4)_2$ (**2**) to illustrate the connectivity (Sn–Br distances above the longest distance in $SnBr_2$ indicated as dotted lines).

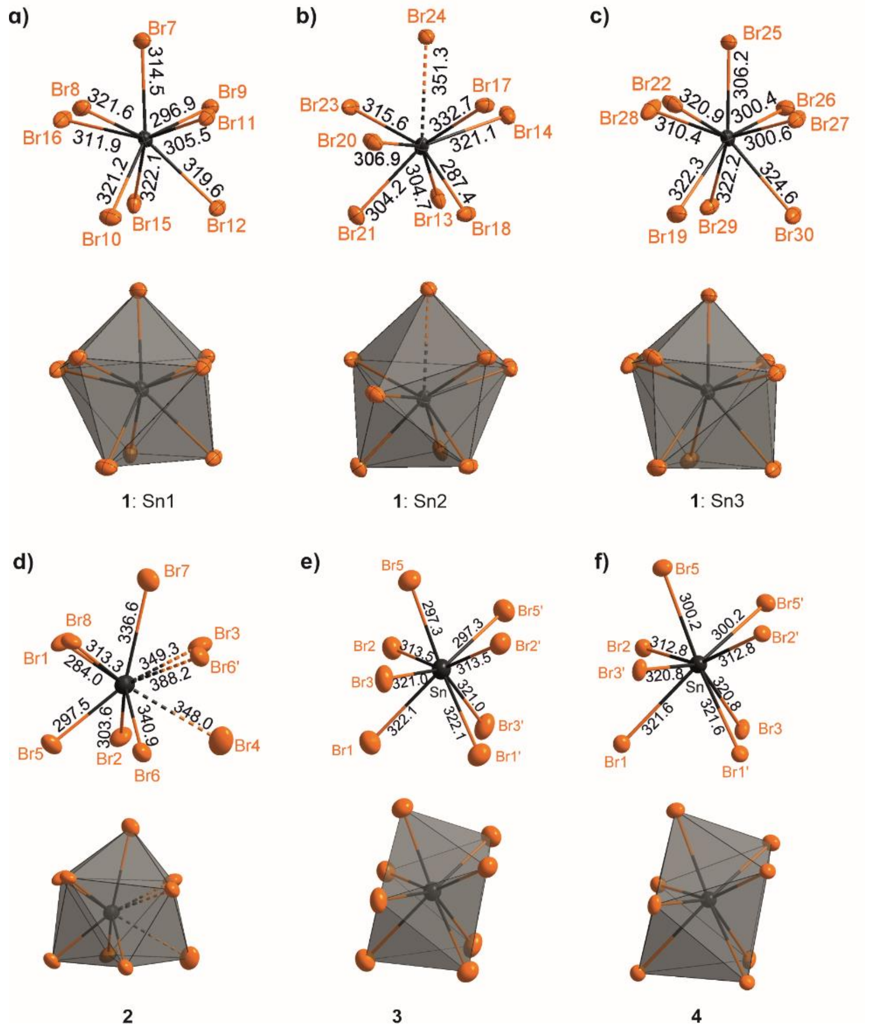

Figure S4. Coordination polyhedra of Sn^{2+} and Sn–Br distances (in pm) in $[Sn_3(AlBr_4)_6](Al_2Br_6)$ (**1**): a) Sn1, b) Sn2, c) Sn3 (c) in comparison to d) $Sn(AlBr_4)_2$ (**2**), e) $[EMIm][Sn(AlBr_4)_3]$ (**3**) and f) $[BMPyr][Sn(AlBr_4)_3]$ (**4**).

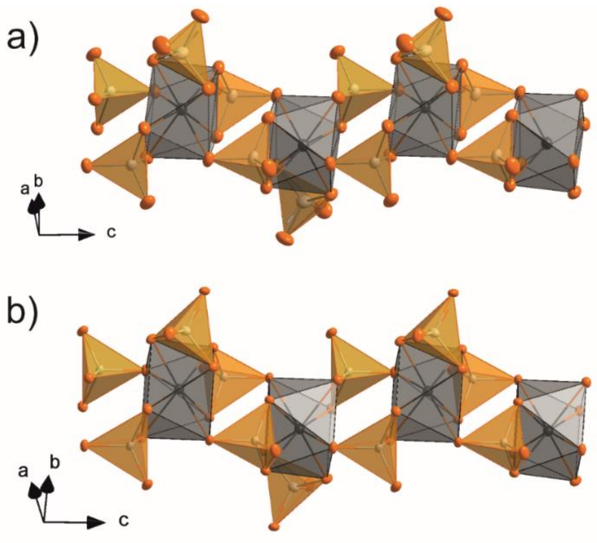

Figure S5. Infinite $_\infty^1[Sn(AlBr_4)_3]^{n-}$ chains in a) [EMIm][$Sn(AlBr_4)_3$] (**3**) and b) [BMPyr][$Sn(AlBr_4)_3$] (**4**) (polyhedra around Sn^{2+}: grey, $[AlBr_4]^-$ tetrahedra: orange).

3. Chemical Composition

To validate the chemical composition of the title compounds, elemental analysis (C,H,N analysis), energy-dispersive X-ray spectroscopy (EDXS) and thermogravimetry (TG) were performed. According to EA, minor amounts of C/H/N are found for **1** and **2**, which can be attributed to adhered ionic liquid on the crystal facets. For **3** and **4**, the values for C and N are slightly higher than calculated, which can be again attributed to adhered ionic liquid on the crystal facets (Table S3). Furthermore, it needs to be noticed that the hydrogen content obtained is too low compared to the calculated values of the [EMIm]/[BMPyr]-containing compounds **3** and **4** (Table S3). We assume the lack of hydrogen is caused by the evaporation of HBr, which cannot be detected with our equipment. To address this limitation, the tin capsules, usually applied to perform EA, were replaced by silver capsules, which prevent HBr evolution due to the formation of AgBr. As a result of EA performed in silver capsules, the obtained H values were indeed higher compared to those obtained with tin capsules (Table S3).

EDXS confirms the presence of Sn and Al/Br (Figure S6a-d). Al and Br cannot be reliably distinguished due to the similar energy of the Al–K_α emission (1.486 eV) and the Br–L_α emission (1.480 eV). Moreover, single crystals of the title compounds decompose rapidly in vacuum under electron bombardment (30 kV) due to Al_2Br_6 release (Figure S6e). As a result, scanning electron microscopy (SEM) after EDXS analysis shows a highly porous remain of a former single crystal. The evaporation of Al_2Br_6 is also confirmed by TG as well as by literature data (sublimation of Al_2Br_6 starting at 28 °C).[S1] Due to total decomposition of the title compounds, TG allows to quantify the chemical composition and to study the thermal properties (*see main paper*, Figure S7).

Table S3. EA of $[Sn_3(AlBr_4)_6](Al_2Br_6)$ (**1**), $Sn(AlBr_4)_2$ (**2**), $[EMIm][Sn(AlBr_4)_3]$ (**3**) and $[BMPyr][Sn(AlBr_4)_3]$ (**4**).

Compound	Type of capsule	C content / %	H content / %	N content / %
$[Sn_3(AlBr_4)_6](Al_2Br_6)$ (**1**)	Sn capsules	0.71	0.08	0.23
	calculated	*0*	*0*	*0*
$Sn(AlBr_4)_2$ (**2**)	Sn capsules	0.60	0.11	0.09
	calculated	*0*	*0*	*0*
$[C_6H_{11}N_2][Sn(AlBr_4)_3]$ (**3**)	Sn capsules	5.85	0.11	2.25
	Ag capsules	5.89	0.43	2.24
	calculated	*5.68*	*0.87*	*2.21*
$[C_9H_{20}N][Sn(AlBr_4)_3]$ (**4**).	Sn capsules	8.65	0.33	1.11
	Ag capsules	8.72	1.29	1.07
	calculated	*8.31*	*1.55*	*1.08*

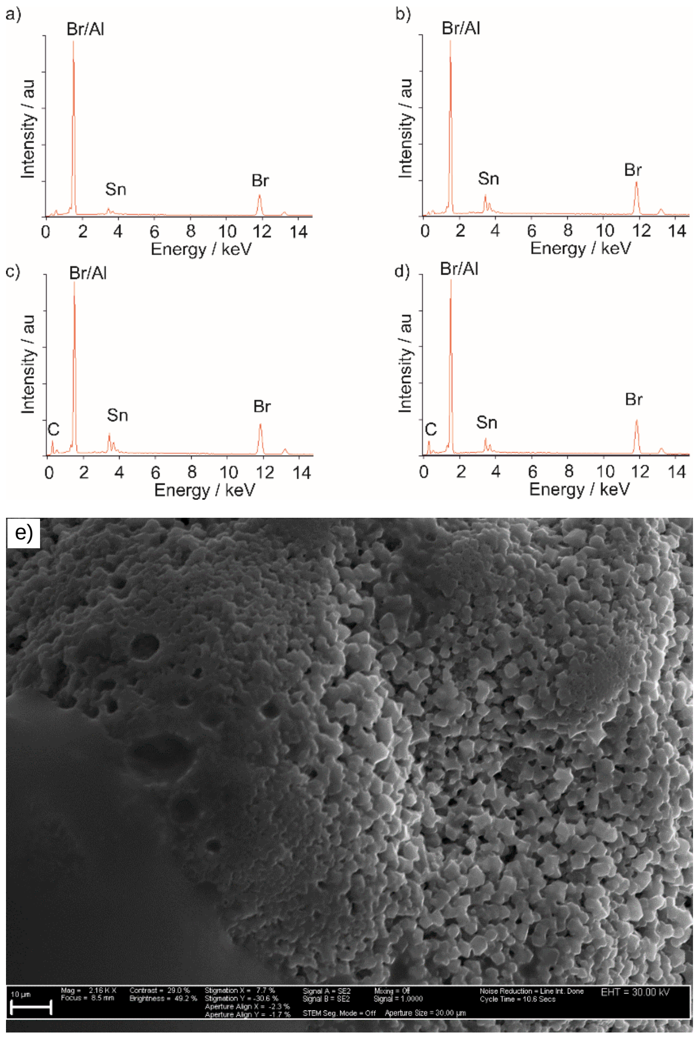

Figure S6. EDXS of a) $[Sn_3(AlBr_4)_6](Al_2Br_6)$ (**1**), b) $Sn(AlBr_4)_2$ (**2**), c) $[EMIm][Sn(AlBr_4)_3]$ (**3**) and d) $[BMPyr][Sn(AlBr_4)_3]$ (**4**) as well as e) SEM image of an exemplary crystal facet of $[Sn_3(AlBr_4)_6](Al_2Br_6)$ (**1**) after EDXS with 5 min electron bombardment at 30 kV.

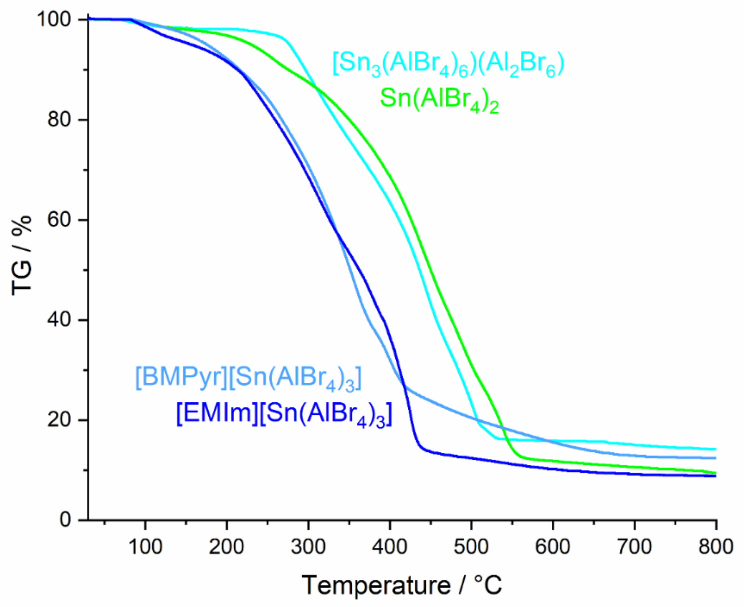

Figure S7. TG of $[Sn_3(AlBr_4)_6](Al_2Br_6)$ (**1**), $Sn(AlBr_4)_2$ (**2**), $[EMIm][Sn(AlBr_4)_3]$ (**3**), $[BMPyr][Sn(AlBr_4)_3]$ (**4**).

In addition, PXRD measurements were tried. However, due to their moisture sensitivity, the samples need to be filled and measured in glass capillaries sealed under argon. Due the low melting, the powders become liquid when mortaring. Even after cooling, the powder particles glue together and form even larger particles. Therefore, we were not able to obtain any suitable diffractograms.

4. Material Properties

To examine the optical properties, ultraviolet-visible (UV-Vis) spectroscopy of all title compounds was performed (Figure S8). Here, absorption below 420 nm (**1**), 450 nm (**2**), and 400 nm (**3, 4**) is observed, which can be related to a Br^{-}-to-Al^{3+} ligand-to-metal charge transfer. The absorption is more-or-less similar to the binary halide $AlBr_3$. Below 250 nm, finally, valence-band to conduction-band absorption occurs.

In regard of the excitation process, in general, there are four options:

(1) Valence-band (V_b) to conduction-band (C_b) excitation: Here, a comparison with the binary phases $SnBr_2$ and $AlBr_3$ is indicative. For $SnBr_2$, $V_b \rightarrow C_b$ excitation is observed < 400 nm (Figure S8). For $AlBr_3$, $V_b \rightarrow C_b$ excitation is observed < 230 nm (Figure S8). In both cases the very intense and very broad absorption points to $V_b \rightarrow C_b$ excitation. The respective wavelengths are in accordance with the literature and not relevant for the excitation of the title compounds.

(2) $Br^{-} \rightarrow Al^{3+}$ LMCT excitation: For $AlBr_3$, $Br^{-} \rightarrow Al^{3+}$ LMCT excitation is observed at 240-370 nm (Figure S8). Shape and position fit very well with all title compounds, which indicates the $Br^{-} \rightarrow Al^{3+}$ LMCT as the origin of the excitation of the title compounds.

(3) Br^- → Sn^{2+} LMCT excitation: The Br^- → Sn^{2+} LMCT excitation is expected to be at significantly higher energy/lower wavelength as compared to the Br^- → Al^{3+} LMCT excitation (due to the lower charge of Sn^{2+} in comparison to Al^{3+}). Therefore, this transition is not relevant here.

(4) *s* → *p* transition on Sn^{2+}: This transition cannot be excluded completely since it is to be expected in a similar wavelength regime as the Br^- → Al^{3+} LMCT excitation. The excitation spectra of the title compound at 240-370 nm, however, look very similar to $AlBr_3$, so the Br^- → Al^{3+} LMCT excitation seems most probable.

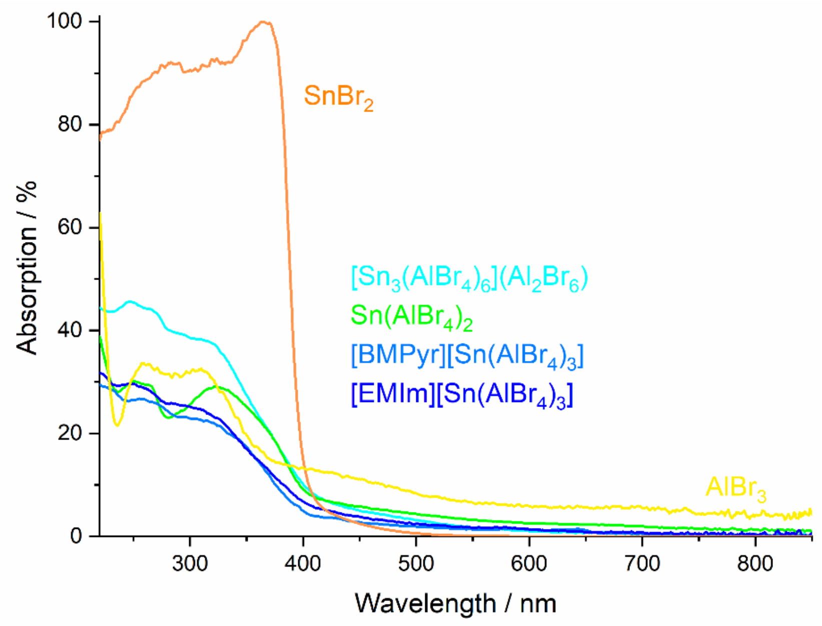

Figure S8. UV-Vis spectra of $[Sn_3(AlBr_4)_6](Al_2Br_6)$ (**1**), $Sn(AlBr_4)_2$ (**2**), $[EMIm][Sn(AlBr_4)_3]$ (**3**) and $[BMPyr][Sn(AlBr_4)_3]$ (**4**) in comparison to $SnBr_2$ and $AlBr_3$ as references.

References

[S1] B. Brunetti, V. Piacente, P. Scardala, *J. Chem. Eng. Data* **2010**, *55*, 2164-2168.
